# Broiler resilience to colibacillosis is affected by incubation temperature and post-hatch feeding strategy

**DOI:** 10.1016/j.psj.2022.102092

**Published:** 2022-08-01

**Authors:** H.J. Wijnen, C.W. van der Pol, A. Papanikolaou, A. Lammers, B. Kemp, H. van den Brand, V. Perricone, M.G.R. Matthijs, R. Molenaar

**Affiliations:** ⁎ Research Department, HatchTech B.V., 3900 AG Veenendaal, the Netherlands; † Adaptation Physiology Group, Department of Animal Sciences, Wageningen University & Research, 6700 AH Wageningen, the Netherlands; ‡ Department of Population Health Sciences, Faculty of Veterinary Medicine, Utrecht University, 3584 CM Utrecht, the Netherlands; § Department of Animal Health and Welfare, Wageningen Livestock Research, 6700 AH Wageningen, the Netherlands

**Keywords:** incubation, eggshell temperature, early feeding, delayed feeding, colibacillosis

## Abstract

Colibacillosis is a poultry disease that negatively affects welfare and causes economic losses. Treatment with antibiotics raises concerns on antimicrobial resistance. Consequently, alternative approaches to enhance poultry resilience are needed. Access to feed and water directly after hatch (**early feeding**) may enhance resilience at later ages. Additionally, a high eggshell temperature (**EST**) during mid incubation may improve chick quality at hatch, supporting potential positive effects of early feeding. Effects of EST [37.8°C (**control**) or 38.9°C (**higher**)] during mid-incubation (embryo days 7–14) and feeding strategy (early feeding or 48 h **delayed feeding**) were tested in a 2 × 2 factorial arrangement. At hatch, ~ 1,800 broilers were divided over 36 pens and grown for 6 wk. At d 8 post hatch, avian pathogenic *E. coli* (APEC) was inoculated intratracheally as model to investigate broiler resilience against respiratory diseases. Incidence and severity of colibacillosis, local infection, and systemic infection were assessed at 6 moments between 3 h and 7 d postinoculation. Broilers were weighed daily during 13 d postinoculation and weekly thereafter. At higher EST, early feeding resulted in higher incidence of systemic infection compared to delayed feeding whereas at control EST, systemic infection was not different between feeding strategies. Regardless of EST, early compared to delayed feeding resulted in lower incidence of local infection, fewer BW deviations, and higher growth until d 35. In conclusion, early feeding could be considered as a strategy to enhance broiler resilience, but only when EST is not too high.

## INTRODUCTION

Colibacillosis is a common poultry disease caused by infection with avian pathogenic *Escherichia coli* (**APEC**). Poultry that suffer from a local APEC infection often show lesions in their respiratory tract and in case of a systemic infection, severe cardiac and hepatic lesions can occur (Gross, 1956). Consequently, poultry health and welfare are impaired and profit is reduced (Yogaratnam, 1995; Matthijs et al., 2017). Live vaccines provide protection against this disease to some extent, but full protection is generally not acquired (Kariyawasam et al., 2004; Rawiwet and Chansiripornchai, 2009; La Ragione et al., 2013; Śmiałek et al., 2021). Treatment with antibiotics can contribute to the rise of antimicrobial resistant *E. coli* strains posing a serious threat for human health (van den Bogaard et al., 2001). Therefore, the poultry industry is searching for alternative approaches to cope with infectious diseases, like colibacillosis, for instance by enhancing animal resilience. Resilience can be defined as the capacity of an animal to deal with environmental disturbances and/or recover with minimal loss of function (Folke et al., 2010). In case the environmental disturbance concerns an infectious disease, a resilient broiler has a lower chance to become infected and, once it does get infected, it will show a milder drop in function (e.g., growth rate) and it will recover faster than a less-resilient broiler.

Resilience of poultry may be enhanced by optimizing perinatal conditions, such as the provision of feed and water directly after hatch, referred to as ‘early feeding’ (Roberts, 1928). In common practice, chicks have first access to feed and water upon arrival at the farm. Upon arrival, chicks are 36 to 48 hours of age or even older due to variation in hatch and pull time, processing, and transport duration (Careghi et al., 2005). Withholding chicks from feed and water during this period seems to result in suboptimal neonatal chick development, shown for instance by a loss in BW and impaired and/or delayed onset of immunocompetence compared to early fed chicks (Noy and Sklan, 1999; Shira et al., 2005; Lamot et al., 2014; Simon et al., 2014; Price et al., 2015; Wijnen et al., 2022). At later ages, early fed broilers showed enhanced growth performance, lower mortality rate, and different immune responses compared to delayed fed broilers (Juul-Madsen et al., 2004; Shira et al., 2005; Lamot et al., 2016; de Jong et al., 2017). Consequently, it can be speculated that early feeding may enhance disease resilience at later age as well. Some indications have been found that early fed broilers have higher resilience to intestinal diseases compared to delayed fed broilers (Dibner et al., 1998; Yi et al., 2005; Ao et al., 2012; Wijnen et al., 2021). However, studies investigating effects of post-hatch feeding strategy on disease resilience are limited and resilience to respiratory diseases, such as colibacillosis has not been studied yet.

De Jong et al. (2017) performed a meta-analysis on 75 papers that studied the effects of post-hatch feeding strategy and demonstrated that considerable variation and inconsistency exists among studies. They speculated that this may be explained, in part, by incubation temperature. Incubation temperature affects embryo development and consequently chick quality at hatch. Too high or too low incubation temperature can negatively affect gut morphology and digestive enzyme activity in newborn chicks (Wineland et al., 2006; Barri et al., 2011; Wijnen et al., 2020b) and it can be speculated that this might result in difficulties to digest and absorb first exogenous feed. Incubation temperature is most accurately reflected by eggshell temperature (**EST**), which in turn reflects embryo body temperature (French, 1997). An increase or decrease in EST will result in an increase or decrease of embryonic metabolism, respectively, as embryos act poikilotherm during the major part of incubation (Romijn and Lokhorst, 1955; Dietz and van Kampen, 1994; French, 1997; Lourens et al., 2006; Szdzuy et al., 2008). Currently, a constant EST of 37.8°C throughout incubation is considered to result in most optimal embryo development and chick quality at hatch (Lourens et al., 2005). However, Nangsuay et al. (2016) showed that an EST of 38.9°C from embryonic day (**E**) 7 onwards resulted in a higher yolk free body mass (**YFBM**) up to E16 compared to a constant 37.8°C EST. This may indicate enhanced embryo body development and chicks may hatch with higher YFBM, less residual yolk, and advanced development of organs, such as the gastrointestinal tract. Therefore, it can be hypothesized that a higher EST of 38.9°C during the second week of incubation advanced chick quality at hatch and that a better chick quality at hatch may enhance the proposed beneficial effects of early feeding on disease resilience.

This study evaluated whether or not broiler resilience to colibacillosis is affected by mid-incubation temperature, post-hatch feeding strategy, and their interaction. Two mid-incubation temperatures (high vs. control) and 2 post-hatch feeding strategies (early vs. delayed) were investigated with a two-by-two factorial design.

## MATERIALS AND METHODS

An experiment was conducted between February and April 2021 at Wageningen University & Research, the Netherlands. All experimental procedures were approved by the Governmental Commission on Animal Experiments, The Hague, the Netherlands, approval number: 2018.W-0020.002.

### Experimental Design

The experiment was set up as a 2 × 2 factorial arrangement with EST during mid incubation and post-hatch feeding strategy as treatments. EST from E7 until E14 was set at 37.8°C (**control**) or at 38.9°C (**higher**). EST was 37.8°C for the remaining incubation periods for both EST treatments. Post-hatch feeding strategy included access to feed and water directly after hatch (**early feeding**) or 48 h after hatch (**delayed feeding**).

### Egg Origin

A batch of hatching eggs from 1 house of a commercial 31-wk-old Ross 308 broiler breeder flock were stored for 3 d at the broiler breeder farm (Boven-Leeuwen, the Netherlands) before transport to a commercial hatchery (Lagerwey BV, Lunteren, the Netherlands). Upon arrival at the hatchery, first the average egg weight of the batch was determined (58.4 g) by bulk weighing 10 egg trays with 150 eggs each. Three equal weight classes were determined within 1.5 g of the average egg weight (56.9–57.9, 57.9–58.9, 58.9–59.9 g). Thereafter, all eggs from the initial batch were weighed individually until 746 first-grade hatching eggs (clean, without hairline cracks or malformations) per weight class were selected (total 2,238 eggs). Eggs of each weight class were equally divided over 30 setter trays (type 150 Setter Tray, HatchTech, Veenendaal, the Netherlands) to exclude potential effects of initial egg weight on the study outcome (Wilson, 1991).

### Incubation

All 30 trays were set in one incubator (PicoClimer setter HT-150, HatchTech, Veenendaal, the Netherlands) directly after indicated egg weighing procedures. Four EST sensors (NTC Thermistors: type DC 95; Thermometrics, Somerset, UK) were attached to the equator of the eggshell of 4 randomly chosen eggs, equally divided over the incubator, using silicone heat sink compound (Type 340; Dow Corning, Midland, MI) and a small piece (approx. 1.5 × 1.5 cm) of elastic permeable tape (Leukotape K, Essity, Hamburg, Germany). A 22-h preincubation warming profile was applied (adapted from van Roovert-Reijrink et al., 2018) meaning that eggs were linearly warmed from room temperature to 27.8°C EST in 5 hours and from 27.8°C to 37.8°C EST in 17 hours. The moment that eggs reached an EST of 37.8°C was considered to be the start of incubation (E0). Until E7, incubator temperature was continuously adjusted, based on the median temperature of the 4 EST sensors to aim at an EST of 37.8°C. Relative humidity was maintained between 50 and 65%.

At E7, all setter trays were equally divided over 4 identical incubators (PicoClimer setter HT-150, HatchTech, Veenendaal, the Netherlands). This procedure took approx. 1 h during which EST dropped to 97°C. Two incubators were set at an EST of 37.8°C (control EST), whereas the other 2 incubators were set at an EST of 38.9°C (higher EST). In the high EST treatment, EST setpoint was linearly increased over a 12-h period. Until E14, EST control in all 4 incubators was performed as described above. Relative humidity was maintained between 40 and 55%.

At E14, all setter trays were collected from the 4 incubators and set in one incubator (PicoClimer setter HT-150, HatchTech, Veenendaal, the Netherlands). This procedure took approx. 1 hour during which EST dropped to 96.5°C. Until E17+17 hours, the EST was set at 37.8°C and controlled as described above. Relative humidity was maintained between 40 and 45%.

During E0 to E17+17 h, eggs were turned every hour by an angle of 35° from horizontal and CO_2_ levels were maintained below 3,500 ppm. At E17+17 hours, all eggs were candled. Clear eggs and eggs containing a dead embryo were opened to determine fertility. Eggs containing a viable embryo (95.8% of fertile eggs at set) were transferred to hatching baskets. Eggs were transferred to one hatcher basket per setter tray and all hatching baskets were set in one incubator (PicoClimer hatcher HT-150, HatchTech, Veenendaal, the Netherlands). Six EST sensors were attached to 6 randomly chosen eggs, equally divided over the incubator, and incubator air temperature set point was manually adjusted if the average of these 6 EST deviated from 37.8°C. After E19+12 h, the incubator temperature was fixed at the actual setting, and EST was allowed to change as chicks started to emerge from the eggshell. Relative humidity was maintained between 30 and 65% and CO_2_ levels were maintained below 2,500 ppm.

### Hatch and Early Feeding

From E19+12 h onwards, every 3 h the incubator was opened to check whether or not chicks had hatched. Any chick that hatched was marked with a colored dot on its head, using a permanent marker. After marking, chicks were placed back in their original hatcher basket to dry. Within 3 to 12 h after a chick was marked, it was pulled from the incubator and classified either as a 2nd grade chick if any abnormality was observed (e.g., crossed beak, blindness, exposed brains, >2 legs, exposed yolk) or as a 1st grade chick (all remaining chicks). All 1st grade chicks were feather sexed, chick quality was determined (see data collection section below), labeled with a plasticized paper neck tag (size 5 × 2 cm), and transferred to hatcher baskets (HatchCare type, HatchTech, Veenendaal, the Netherlands). All baskets were stored in one section of a HatchCare unit (HatchTech Veenendaal, the Netherlands) until E21+13 h. In half of these baskets (early feeding treatment), ad libitum starter diet (diet details provided in ‘diet and vaccinations’ section below) and fresh water were provided. The other half of the baskets had empty water gutters and feeding troughs (delayed feeding treatment). Relative humidity was maintained between 25 and 75% and CO_2_ levels were maintained below 2,000 ppm. At E21+13 h, all baskets were transported in a climate-controlled van designed for chick transportation (Chickliner, Renswoude, the Netherlands) for approximately 30 min to the grow out facilities (Wageningen University, Wageningen, the Netherlands).

### Grow Out

#### Layout

Upon arrival at the grow-out facility (regarded as d 0), all 1st grade chicks were randomly allocated to 36 floor pens (9 replicate pens / treatment), accounting for the treatment and the sex (equal sex ratio within pen), resulting in 52 to 58 chicks per pen. Pens were located in 3 neighboring rooms (12 pens / room) and within each room, 3 blocks of 4 pens each were formed. Treatments were randomly allocated within a block. Pen size was 200 × 100 cm, contained 7 drinking nipples with drip cups, and had one feeder pan. The concrete pen floor was completely covered with a thin (approx. 1 cm) layer of wood shavings. Broilers were grown for 6 wk.

#### Delayed Feeding

Pens from the delayed feeding treatment were temporarily divided with hardboard into an unfed side and a fed side. Furthermore, in the delayed fed pens, all drinking nipples were temporarily removed and a stand-alone drinker was provided in the fed side. Sides were out of sight from each other. All broilers belonging to the delayed feeding groups were first placed in the unfed side of the pen and each broiler was relocated individually to the fed side of the pen between 48 and 54 h after it had hatched. All broilers within the early fed pens had access to the entire pen from placement onwards. Additionally, at placement, the wood shavings of all 36 pens were covered with cardboard to prevent any litter consumption in the delayed feeding treatment. Hardboard (delayed fed pens) and cardboard (all pens) were removed once the last broiler was relocated to the fed side and stand-alone drinkers were exchanged for drinking nipples (delayed fed pens). Pen treatment was blinded from that moment onwards.

#### Diet and Vaccinations

Once fed, feed and water were provided ad libitum. A starter diet was provided from hatch until d 10, a grower diet from d 10 until 24, and a finisher diet from d 24 onward (Table 1). All diets were pelleted with a diameter 3.0 to 3.3 mm, did not contain coccidiostats, and were produced by Research Diet Services (**RDS**, Wijk bij Duurstede, the Netherlands) according to the guidelines of the Federation Dutch Animal Feed chain (CVB, 2016). Broilers were not vaccinated at the hatchery. At d 29, infectious bronchitis vaccine (Nobilis IB Ma5, batch A267B1J01, MSD) buffered in sterile blue colored solvent (batch G554A02, MSD) was administered via a droplet in one eye and a droplet in one nostril.

**Table 1:** Ingredients and nutrient contents of starter, grower, and finisher diet.

	Starter	Grower	Finisher
Ingredient (g / kg)			
Corn	343.15	224.5	155.45
Soybeanmeal	300	245	200
Wheat	250	400	500
Rapeseedmeal	40	50	60
Lime fine	17.50	14	13
Soybean oil	28	18	16
Monocalciumphophate	7	4	1.5
Premix mais	5	5	5
NaHCO3	2.40	2.1	2
Salt	2.2	2.1	1.9
DL-methionine	2.2	2	1.7
L-lysine HCL	1.8	2.2	2.35
L-Threonine	0.5	0.7	0.7
L-valine	0.15	0.3	0.3
Axtra PHY	0.05	0.05	0.05
Ronozyme WX5000CT	0.05	0.05	0.05
Animal fat (lard)	-	30	40
Calculated composition			
Metabolizable energy broiler (MJ / kg)	2,850	2,950	2,999
Crude protein (g / kg)	216.5	201.3	188.8
Dig. lysine (g / kg)	11.01	10.25	9.51
Calcium (g / kg)	9.0	7.2	6.4
Dig. methionine (g / kg)	5.08	4.68	4.23
Dig. phosphorus poultry (g / kg)	4.2	3.6	3.1

#### Housing

Early and delayed fed broilers can differ in their preferred ambient temperature (Wijnen et al., 2022). To account for this, an ambient temperature gradient was created by a relatively low whole-house setpoint at placement of 28°C and the provision of a heat lamp (100 W infrared incandescent PAR38) at approx. 15 cm above broiler height (adjusted manually with age) in the center of each pen, meaning that each broiler could choose its own preferred ambient temperature. Whole-house setpoint was linearly decreased to 18°C at d 27, and this setpoint was maintained until the end of the study. Relative humidity setpoint was 60 to 70% up to 3 d of age and 50 to 60% thereafter. Continuous light was provided until d 2, and from d 2 onward, 1 h of darkness was added each day until a light schedule of 6 h darkness: 18 h light was provided by d 7 and onward.

### Disease Model

Clinical respiratory colibacillosis was induced by adapting a disease model from Ask et al. (2006a). At day eight, 34 broilers of both sexes (equal ratio) were randomly selected within each pen for *E. coli* inoculation whereas the remaining broilers within each pen (n = 18–24) were selected for placebo inoculation. Horizontal transmission between *E. coli* and placebo inoculated broilers does not occur (Berghof et al., 2019b). Broilers were inoculated intratracheally with either 0.3 mL phosphate buffered saline (**PBS**) for the placebo broilers, or 0.3 mL PBS containing avian pathogenic *Escherichia coli* serotype O78:K80 strain 506 (**APEC**) to induce colibacillosis. The inoculation was performed using a blunted anal cannula fitted on a 1.0-mL syringe. The APEC originated from a frozen culture (−70°C) that had previously been isolated from an inflamed pericardium of a commercial broiler suffering from natural colibacillosis (Van Eck and Goren, 1991). The inoculum was prepared as described by Matthijs et al. (2003), which resulted in 1.53 × 10^7^ CFU/mL (determined by the Veterinarian Microbiological Diagnostic Centre, Utrecht University, Utrecht, the Netherlands). All intratracheal inoculations were performed by trained personnel.

### Data Collection

Incubation duration was calculated as the number of hours from E0 (start of incubation) to emergence from the eggshell. Chick quality from all 1st grade chicks was determined by measuring BW, chick length from beak-tip to toe-tip, and navel score according to the protocol of Reijrink et al. (2009). Additionally, every 36th chick per EST treatment that hatched was euthanized through decapitation until 25 chicks per EST treatment were collected. Residual yolk (**RY**) and heart were weighed on a 3-decimal scale and YFBM was calculated as BW minus RY weight. Relative heart weight was calculated as heart weight divided by YFBM times 100.

Disease morbidity was assessed by observation of lesions at 6 time points post inoculation (**p.i.**) (3 h, 12 h, 1 d, 2 d, 4 d, and 7 d). At each time point, 2 APEC inoculated broilers per pen (1 female and 1 male) were randomly selected for dissection. At 7 d p.i., 8 additional APEC inoculated broilers per pen (equal sex ratio) were randomly selected for dissection. At each time point, the selected broilers were euthanized by decapitation and the presence of lesions in left and right thoracic air sac, pericardium, and serosal surface of the liver were macroscopically assessed. Lesions of each organ were scored 0 (no lesions), 0.5 (a single pinhead-sized inflammatory spot), 1 (two or more pinhead-sized spots), 2 (fibrinous patches on various locations), or 3 (extensive fibrination and exudation) according to the protocol of Van Eck and Goren (1991) by trained poultry veterinarians. The sex of all dissected birds was verified by checking the gonads.

Subsequently, incidences of total lesions, local lesions, and systemic lesions were determined as follows. For incidence of total lesions, all four lesion scores were summed and if the sum was > 0, the broiler was classified as colibacillosis positive. For incidence of local lesions, both air sac lesion scores were summed and in if the sum > 0, the broiler was classified local lesions positive. For incidence of system lesions, lesion scores of the pericardium and liver were summed and if the sum > 0, the broiler was classified systemic lesions positive.

Severity of lesions was evaluated by calculating total mean lesion score (**tMLS**), local mean lesion score (**lMLS**), and systemic mean lesions score (**sMLS**) as follows. tMLS was calculated for each broiler that was classified colibacillosis positive by the sum of all four lesion scores. lMLS was calculated for each broiler that was classified local lesions positive by the sum of both air sac lesion scores. sMLS was calculated for each broiler that was classified systemic lesions positive by the sum of pericardium lesion score and liver lesion score. Severity classifications will be explained in the statistical analysis section.

The presence of *E. coli* in air sacs (local infection) and blood (systemic infection) was determined in 2 broilers / pen for each time point, randomly selected among the dissected animals, according to an adapted protocol from Cuperus et al. (2016). For isolation of *E. coli* from blood, right before decapitation a part of the skin above the wing vein was disinfected and approximately 1 mL of blood was collected with single use needles and syringes and without any anticoagulant. Immediately thereafter, three droplets of blood were dripped on a McConkey agar plate (Balis, Boven-Leeuwen, the Netherlands) and spread with a disposable spreader, using the spread plate technique. For isolation of *E. coli* from air sacs, the thoracic air sac with the most severe lesions was swabbed immediately after postmortem examination using transport swabs with Amies medium (Uni-ter CLR 230397 lot 30380, Meus, Piove di Sacco, Italy). The left air sac was swabbed in case lesion severity was similar between left and right air sac or in case no lesions occurred. Swabs with Amies medium were stored in a fridge (~ 7°C) until the next day and then spread on McConkey agar using a streak technique. Bacterial growth was evaluated after overnight incubation at 37.5°C by counting the number of colony-forming units (**CFU**). Incidence of *E. coli* in air sacs and blood was calculated by classifying agar plates with > 0 CFU as *E. coli* positive. Amount of *E. coli* was determined by counting the number of CFU for each agar plate that was classified as *E. coli* positive.

All broilers were individually weighed daily during 13 days p.i., except at 7 days and 12 days p.i.. Broilers that died or were dissected during these 13 d were only included in analysis if BW could be recorded for at least 5 consecutive days during these 13 days p.i. (n = 806 broilers). For each day p.i., standardized BW deviation was determined by comparing the standardized BW from each APEC inoculated broiler to the average BW of placebo inoculated broilers from the same sex and treatment. ‘Standardized BW’ was BW that was standardized to standardized deviation of corresponding treatment group and day p.i. to correct for scaling differences in standardized deviations between ages and treatment groups. The natural logarithm of the variance (**LNvar**), skewness, and lag-one autocorrelation of standardized BW deviations were calculated as resilience indicators according to a protocol of Berghof et al. (2019a). Additionally, all broilers were weighed individually every week to observe growth performance until the end of the experiment (d 42).

Survival was monitored during 34 days p.i.. During the first 3 days p.i., mortality was checked every 3 h. During the remaining period, mortality was checked daily. Broilers were culled if a humane endpoint was reached as described by Berghof et al. (2019b). Moment of death or cull was noted, and carcasses were saved in a freezer for necropsy at the end of the experiment. Necropsy was performed by a poultry veterinarian to determine suspected cause of death. Causes of death were divided into colibacillosis (septic hemorrhage in organs or lesions in air sacs, pericardium, and/or liver) or other reasons than the *E. coli* infection.

### Statistical Analyses

All data were analyzed using the statistical software package SAS (Version 9.4, SAS Institute, 2010). A *P*-value < 0.05 was considered to be significant, and *P*-values >0.05 and <0.10 were considered to be a tendency. The model used for all data at hatch was

(1) $$Y_{\text{ij}}=\mu +\text{ES}T_{i}+\text{SE}X_{j}+\text{EST}\times \text{SE}X_{\text{ij}}+e_{\text{ij}}$$

where Y_ij_ =the dependent variable, μ=the overall mean, EST_i_ = eggshell temperature during mid-incubation (_i_ = 37.8 or 38.9°C), SEX_j_ = sex (_j_ = female or male), EST × SEX_ij_ = the interaction between EST and SEX, and e_ij_ = the error term. Hatching basket was considered to be the experimental unit by adding hatching basket nested within incubator as a random factor. The EST × SEX interactions was excluded from the model when *P* > 0.05. The PROC MIXED procedure was used to analyze incubation duration, BW, RY, YFBM, chick length and relative heart weight. Model assumptions were verified by inspection of ‘raw residuals vs predicted values’ plot and ‘Q-Q’ plot of the residuals and skewness and kurtosis between −2 to +2. All data were normally distributed and presented as LSmeans ± SEM. The PROC GLIMMIX procedure was used to analyze navel score, using a multinomial distribution, and a cumlogit link function. Navel score is presented as mean ± SE.

The basic model used for all post hatch data was

(2) $$Y_{\text{ijk}}=\mu +\text{ES}T_{i}+\text{FEE}D_{j}+\text{EST}\times \text{FEE}D_{\text{ij}}+\text{SE}X_{k}+\text{EST}\times \text{SE}X_{\text{ik}}+\text{FEED}\times \text{SE}X_{\text{jk}}+\text{EST}\times \text{FEED}\times \text{SE}X_{\text{ijk}}+e_{\text{ijk}}$$

where Y_ijk_ = the dependent variable, μ = the overall mean, EST_i_ = eggshell temperature during mid-incubation (_i_ = 37.8 or 38.9°C), FEED_j_ = feeding strategy (_j_ = early or delayed), EST × FEED_ij_ = the interaction between EST and FEED, SEX_k_ = sex (_k_ = female or male), EST × SEX_ik_ = the interaction between EST and SEX, FEED × SEX_jk_ = the interaction between FEED and SEX, EST × FEED × SEX_ijk_ = the 3-way interaction between EST and FEED and SEX, and e_ijk_ = the error term. Pen was considered to be the experimental unit by adding pen (1–36) nested within block (1–9) as a random factor. Treatment × SEX interactions were excluded from the model when *P* > 0.05.

The PROC GLIMMIX procedure was used to analyze incidence of colibacillosis, incidence of local lesions, incidence of systemic lesions, incidence of *E. coli* in air sacs, incidence of *E. coli* in blood, tMLS, lMLS, sMLS, amount of *E. coli* in air sacs, and amount of *E. coli* in blood. tMLS was divided into 6 equal classes (0.5–2 = class 1, 2.5–4 = class 2, 4.5–6 = class 3, 6.5–8 = class 4, 8.5–10 = class 5, >10 = class 6), lMLS into 5 equal classes (0.5–1 = class 1, 1.5–2 = class 2, 2.5–3 = class 3, 3.5–4 = class 4, >4 = class 5), and sMLS into 5 equal classes (0.5–1 = class 1, 1.5–2 = class 2, 2.5–3 = class 3, 3.5 4 = class 4, >4 = class 5). Dissection moment was added to model 2 as a fixed factor. All incidences were analyzed with a binary distribution and a logit link function in model 2. tMLS, lMLS, and sMLS were analyzed with a multinomial distribution and a cumulative logit link function in model 2. Amount of *E. coli* in air sacs and *E. coli* in blood were analyzed with a Poisson log link function in model 2 with dissection moment added as a fixed factor. Data are expressed as mean ± SE.

The PROC MIXED procedure was used to analyze weekly BW (separately for each week) and LNvar, skewness, and lag-one autocorrelation of standardized BW deviations. Model assumptions were verified as previously indicated. All data were normally distributed. Data are expressed as LSmeans ± SEM.

The PROC PHREG procedure (cox proportional hazard model) was used to perform a survival analysis on APEC inoculated broilers for the period p.i. (>7 d). Broilers that were dissected or euthanized at the end of the experiment were censored. Broilers that were suspected during necropsy to have died or culled due to other reason than APEC were excluded from analysis (n = 10). Block was added as random factor. Model assumptions were verified by a supremum test (Kleinbaum and Klein, 2012).

## RESULTS

### Chick Quality at Hatch

Chick length at hatch showed an interaction between EST × sex. Control and higher EST females did not differ in length, whereas control EST males were shorter than higher EST males (∆ = 0.1 cm; *P* = 0.02; Table 2). Higher EST had shorter incubation duration compared to control EST (∆ = 4 h; *P* < 0.0001). Chick BW, RY, YFBM, relative heart weight, and navel score did not differ between EST groups (*P* ≥ 0.26).

**Table 2:** Effect of eggshell temperature (EST) during mid incubation^1^ on incubation duration and chick quality characteristics at hatch moment.

Treatment	n^2^	Duration (h)	Chick weight (g)	Residual yolk weight (g)	Yolk-free body mass (g)	Chick length^3^ (cm)	Navel condition^4^ (score)	Heart^5^ weight (%)
EST								
Control	15	487^a^	43.02	5.81	37.33	18.6	1.5 ± 0.02	0.82
Higher	15	483^b^	43.10	5.81	37.05	18.7	1.5 ± 0.02	0.81
SEM		0.4	0.052	0.237	0.251	0.02	-	0.022
*P*-values								
EST		<0.0001	0.26	0.99	0.45	<0.01	0.92	0.83
EST × Sex		-	-	-	-	0.02^6^	-	-

Note: Data are presented as least square mean ± SEM, except for navel condition (mean ± SE).

1 EST during mid incubation (embryo days 7–14) was either 37.8°C (**Control**) or 38.9°C (**Higher**), and the remaining incubation period EST was 37.8°C for both treatment groups.

2 Trays nested in incubator.

3 Body length from beak-tip to toe-tip.

4 Navel condition: score 1 (perfect), 2 (discolored/opened < 2 mm), 3 (discolored/opened > 2 mm).

5 Weight relative to yolk-free body mass.

6 Length was 18.7^ab^, 18.6^c^, 18.8^a^, and 18.7^b^ cm for control females, control males, higher females, higher males respectively.

a-b Least square means within a column lacking a common superscript differ (*P* < 0.05).

### Colibacillosis

Incidence of colibacillosis did not show an interaction between EST and feeding strategy (*P* = 0.67; Table 3), nor a main effect of EST (*P* = 0.26). Incidence of colibacillosis tended to be lower for early fed broilers compared to delayed fed broilers (∆ = 2 %; *P* = 0.09). tMLS showed an interaction between EST and feeding strategy (*P* < 0.01). At control EST, tMLS was higher in delayed fed broilers than early fed broilers (∆ = 0.8 lesion score), whereas the opposite was found at higher EST (∆ = 0.7 lesion score).

**Table 3:** Effect of eggshell temperature (EST) during mid-incubation^1^ and post-hatch feeding strategy^2^ on incidence and severity of colibacillosis lesions^3^ in broilers during colibacillosis^4^.

Treatment	n^5^	No lesions^6^ (%)	tMLS^7^ (score)
EST			
Control	18	38 ± 2.6	3.0 ± 0.25
Higher	18	37 ± 2.5	2.9 ± 0.23
Feeding strategy			
Delayed	18	37 ± 2.5	2.9 ± 0.24
Early	18	39 ± 2.6	2.9 ± 0.24
EST × Feeding strategy			
Control × Delayed	9	34 ± 3.6	3.4 ± 0.37^a^
Control × Early	9	41 ± 3.7	2.6 ± 0.32^b^
Higher × Delayed	9	39 ± 3.5	2.5 ± 0.29^b^
Higher × Early	9	36 ± 3.6	3.2 ± 0.34^a^
*P*-values			
EST		0.26	0.90
Feeding strategy		0.09	0.77
EST × Feeding strategy		0.67	<0.01

Note: Data are presented as mean ± SE.

1 EST during mid-incubation (embryo days 7–14) was either 37.8°C (**Control**) or 38.9°C (**Higher**), and the remaining incubation period EST was 37.8°C for both treatment groups.

2 Feeding strategy was either direct access to feed and water after hatch (**Early**) or 48 h after hatch (**Delayed**).

3 Sum of lesion scores from left air sac, right air sac, pericardium, and liver, each scored 0 (clean), 0.5 (single spot), 1 (two or more spots), 2 (patches), or 3 (extensive fibrination).

4 Colibacillosis was induced by intratracheal *E. coli* (O78:K80 strain 506) inoculation at d 8 of age (dose 0.3 mL of 1.53 × 10^7^ CFU/mL).

5 Pens, with 20 broilers dissected / pen, divided over 6 moments post inoculation (3 and 12 h, 1-2-4-7 days).

6 Colibacillosis lesions^3^ = 0.

7 tMLS = total Mean Lesion Score from broilers with colibacillosis lesions^3^ > 0.

a-b Means within a column and factor lacking a common superscript differ (*P* < 0.05).

### Local *E. coli* Infection

Incidence and amount of *E. coli* in air sacs did not show an interaction between EST and feeding strategy (*P* ≥ 0.31; Table 4), nor a main effect of EST (*P* ≥ 0.18) or feeding strategy (*P* ≥ 0.57). Incidence of local lesions did not show an interaction between EST and feeding strategy (*P* = 0.55), nor a main effect of EST (*P* = 0.20). Incidence of local lesions was lower for early fed broilers compared to delayed fed broilers (∆ = 6 %; *P* = 0.046). lMLS did not show an interaction between EST and feeding strategy (*P* = 0.24), nor a main effect of EST (*P* = 0.93) or feeding strategy (*P* = 0.42).

**Table 4:** Effect of eggshell temperature (EST) during mid incubation^1^ and post-hatch feeding strategy^2^ on incidence and severity of *E. coli* in air sac^3^ and local lesions^4^ in broilers during colibacillosis^5^.

Treatment	n^6^	*E. coli* in air sac		Local lesions	
		Incidence^7^ (%)	Amount^8^ (CFU)	Incidence^9^ (%)	Severity^10^ (lMLS)
EST					
Control	18	34 ± 3.2	121 ± 81.2	61 ± 2.6	1.9 ± 0.12
Higher	18	41 ± 3.3	100 ± 62.2	65 ± 2.5	1.9 ± 0.12
Feeding strategy					
Delayed	18	39 ± 3.3	132 ± 75.1	66 ± 2.5^a^	1.9 ± 0.12
Early	18	36 ± 3.3	88 ± 65.2	60 ± 2.6^b^	1.9 ± 0.12
EST × Feeding strategy					
Control × Delayed	9	38 ± 4.6	159 ± 120.3	65 ± 3.6	2.1 ± 0.18
Control × Early	9	30 ± 4.5	81 ± 101.8	56 ± 3.7	1.7 ± 0.17
Higher × Delayed	9	40 ± 4.7	104 ± 91.2	67 ± 3.5	1.8 ± 0.15
Higher × Early	9	42 ± 4.8	95 ± 85.8	63 ± 3.6	2.0 ± 0.18
*P*-values					
EST		0.18	0.37	0.20	0.93
Feeding strategy		0.57	0.94	0.05	0.42
EST × Feeding strategy		0.31	0.55	0.55	0.24

Note: data are presented as mean ± SE.

1 EST during mid-incubation (embryo days 7–14) was either 37.8°C (**Control**) or 38.9°C (**Higher**), and the remaining incubation period EST was 37.8°C for both treatment groups.

2 Feeding strategy was either direct access to feed and water after hatch (**Early**) or 48 h after hatch (**Delayed**).

3 *E. coli* colony forming units (**CFU**) from air sac swab plated on McConkey-agar.

4 Sum of lesion scores from left and right air sac, each scored 0 (clean), 0.5 (single spot), 1 (two or more spots), 2 (patches), or 3 (extensive fibrination).

5 Colibacillosis was induced by intratracheal *E. coli* (O78:K80 strain 506) inoculation at d 8 of age (dose 0.3 mL of 1.53 × 10^7^ CFU/mL).

6 Pens, with 20 broilers dissected / pen, divided over 6 moments post inoculation (3 and 12 h, 1-2-4-7 days).

7 Classified ‘Incidence positive’ if CFU^3^ > 0.

8 From *E. coli* in air sac incidence positive broilers^7^.

9 Classified ‘Incidence positive’ if local lesion score^4^ >0.

10 lMLS = local Mean Lesion Score from local lesion incidence positive broilers^9^.

a-b Means within a column and factor lacking a common superscript differ (*P* < 0.05).

### Systemic *E. coli* Infection

Incidence of *E. coli* in blood (*P* = 0.01) and incidence of systemic lesions (*P* = 0.03) both showed and interaction between EST and feeding strategy (Table 5). At control EST, no effect of feeding strategy was found, but at higher EST, delayed fed broilers had a lower incidence of *E. coli* in blood and a lower incidence of systemic lesions than early fed broilers (∆ = 11% and Δ = 10%, for *E. coli* in blood and systemic lesions, respectively). Amount of *E. coli* in blood did not show an interaction between EST and feeding strategy (*P* = 0.99), nor a main effect of EST (*P* = 0.71) or feeding strategy (*P* = 0.49). sMLS did not show an interaction between EST and feeding strategy (*P* = 0.45), nor a main effect of EST (*P* = 0.83) or feeding strategy (*P* = 0.67).

**Table 5:** Effect of eggshell temperature (EST) during mid incubation^1^ and post-hatch feeding strategy^2^ on incidence and severity of *E. coli* in blood^3^ and systemic lesions^4^ in broilers during colibacillosis^5^.

Treatment	n^6^	*E. coli* in blood		Systemic lesions	
		Incidence^7^ (%)	Amount^8^ (CFU)	Incidence^9^ (%)	Severity^10^ (sMLS)
EST					
Control	18	20 ± 2.7	10 ± 16.9	28 ± 2.4	1.1 ± 0.24
Higher	18	18 ± 2.6	17 ± 50.7	27 ± 2.4	1.0 ± 0.22
Feeding strategy					
Delayed	18	18 ± 2.6	16 ± 51.5	26 ± 2.3	1.0 ± 0.25
Early	18	20 ± 2.8	11 ± 14.9	29 ± 2.4	1.1 ± 0.21
EST × Feeding strategy					
Control × Delayed	9	23 ± 4.0^a^	13 ± 24.3	30 ± 3.4^ab^	1.3 ± 0.34
Control × Early	9	17 ± 4.0^ab^	8 ± 22.8	26 ± 3.3^ab^	1.0 ± 0.32
Higher × Delayed	9	13 ± 3.2^b^	20 ± 141.9	22 ± 3.1^b^	0.8 ± 0.36
Higher × Early	9	24 ± 4.1^a^	14 ± 19.9	32 ± 3.5^a^	1.3 ± 0.28
*P*-values					
EST		0.54	0.71	0.89	0.83
Feeding strategy		0.42	0.49	0.38	0.67
EST × Feeding strategy		0.01	0.99	0.03	0.45

Note: data are presented as mean ± SE.

1 EST during mid-incubation (embryo days 7–14) was either 37.8°C (**Control**) or 38.9°C (**Higher**), and the remaining incubation period EST was 37.8°C for both treatment groups.

2 Feeding strategy was either direct access to feed and water after hatch (**Early**) or 48 h after hatch (**Delayed**).

3 *E. coli* colony forming units (**CFU**) from blood swab plated on McConkey-agar.

4 Sum of lesion scores from pericardium and liver, each scored 0 (clean), 0.5 (single spot), 1 (two or more spots), 2 (patches), or 3 (extensive fibrination).

5 Colibacillosis was induced by intratracheal *E. coli* (O78:K80 strain 506) inoculation at d 8 of age (dose 0.3 mL of 1.53 × 10^7^ CFU/mL).

6 Pens, with 20 broilers dissected / pen, divided over 6 moments post inoculation (3 and 12 h, 1-2-4-7 days).

7 Classified ‘Incidence positive’ if CFU^3^ ≥1.

8 From *E. coli* in blood incidence positive broilers^7^.

9 Classified ‘Incidence positive’ if systemic lesion score^4^ >0.

10 sMLS = systemic Mean Lesion Score from systemic lesion incidence positive broilers^9^.

a-b Means within a column and factor lacking a common superscript differ (*P* < 0.05).

### Body Weight

**Figure 1:**
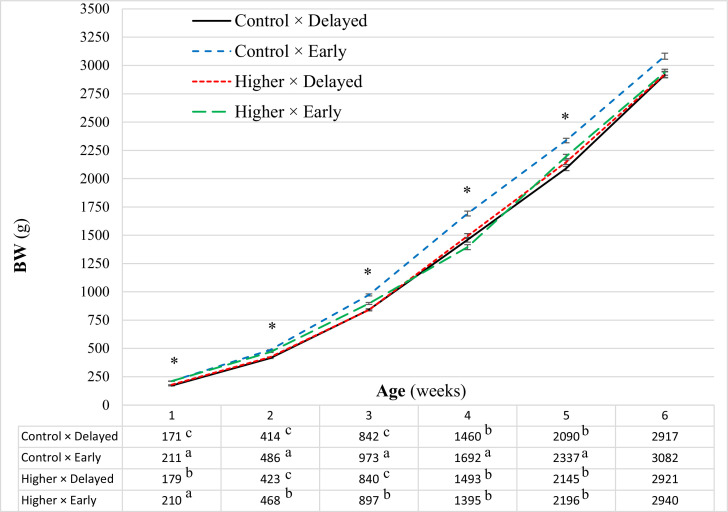
Effect of the interaction between eggshell temperature (37.8°C [**Control**] or 38.9°C [**Higher**]) during mid-incubation (embryo days 7–14) with post-hatch feeding strategy (direct access to feed and water after hatch [**Early**] or 48 h deprivation [**Delayed**]) on broiler BW weekly post avian pathogenic *E. coli* inoculation performed at d 8. Data are presented as LSMean. Error bars indicate SEM. * indicates significant interaction between EST and feeding strategy. ^abc^ indicates least square means within a week lacking a common superscript differ significant. Significant = *P* < 0.05.

Body weights showed an interaction between EST and feeding strategy at all weeks measurements (*P* ≤ 0.03; Figure 1), except for wk 6 (*P* = 0.17). At wk 1, one day prior to inoculation, early fed broilers incubated at higher EST or control EST had highest BW, delayed fed broilers incubated at higher EST had intermediate BW, and delayed fed broilers incubated at control EST had lowest BW. At wk 2 and 3, early fed broilers incubated at control EST had highest BW, early fed broilers incubated at higher EST had intermediate BW, and delayed fed broilers incubated either at control EST or higher EST had lowest BW. At wk 4 and 5, early fed broilers incubated at control EST had higher BW compared to the other treatment groups which were similar to each other. At wk 6, early fed broilers tended to have higher BW compared to delayed fed broilers (∆ = 92 g; *P* = 0.08). EST had no effect on BW at wk 6 (*P* = 0.19).

LNvar, skewness, and lag-one autocorrelation of standardized BW deviations during 13 d p.i. did not show an interaction between EST and feeding strategy (*P* ≥ 0.12; Table 6), nor a main effect of EST (*P* ≥ 0.19). LNvar was lower for early fed broilers compared to delayed fed broilers (∆ = 0.27; *P* = 0.02), whereas skewness and lag-one autocorrelation did not differ between feeding strategies (*P* ≥ 0.19).

**Table 6:** Effect of eggshell temperature (EST) during mid incubation^1^ and post-hatch feeding strategy^2^ on standardized deviations of BW^3^ from broilers during 13 d post inducing colibacillosis^4^.

Standardized BW deviations				
	n^5^	LNvar	Skewness	Lag-one autocorrelation
EST				
Control	18	−2.55	−0.31	0.32
Higher	18	−2.70	−0.28	0.30
SEM		0.081	0.04	0.013
Feeding strategy				
Delayed	18	−2.49^a^	−0.33	0.31
Early	18	−2.76^b^	−0.25	0.31
SEM		0.082	0.04	0.013
EST × Feeding strategy				
Control × Delayed	9	-2.51	−0.36	0.31
Control × Early	9	−2.59	−0.25	0.33
Higher × Delayed	9	−2.48	−0.30	0.30
Higher × Early	9	−2.92	−0.26	0.30
SEM		0.12	0.06	0.019
*P*-values				
EST		0.19	0.67	0.35
Feeding strategy		0.02	0.19	0.79
EST × Feeding strategy		0.12	0.57	0.64

Note: Data are presented as least square mean ± SEM.

1 EST during mid-incubation (embryo day 7–14) was either 37.8°C (**Control**) or 38.9°C (**Higher**), and the remaining incubation period EST was 37.8°C for both treatment groups.

2 Feeding strategy was either direct access to feed and water after hatch (**Early**) or 48 h after hatch (**Delayed**).

3 Deviation of BW compared to placebo. Standardized and calculated as provided in Material and Methods section.

4 Colibacillosis was induced by intratracheal *E. coli* (O78:K80 strain 506) inoculation at day 8 of age (dose 0.3 mL of 1.53 × 10^7^ CFU/mL).

5 Pens (approx. 34 broilers observed / pen)

a-b Least square means within a column and factor lacking a common superscript differ (*P* < 0.05).

### Survival

**Figure 2:**
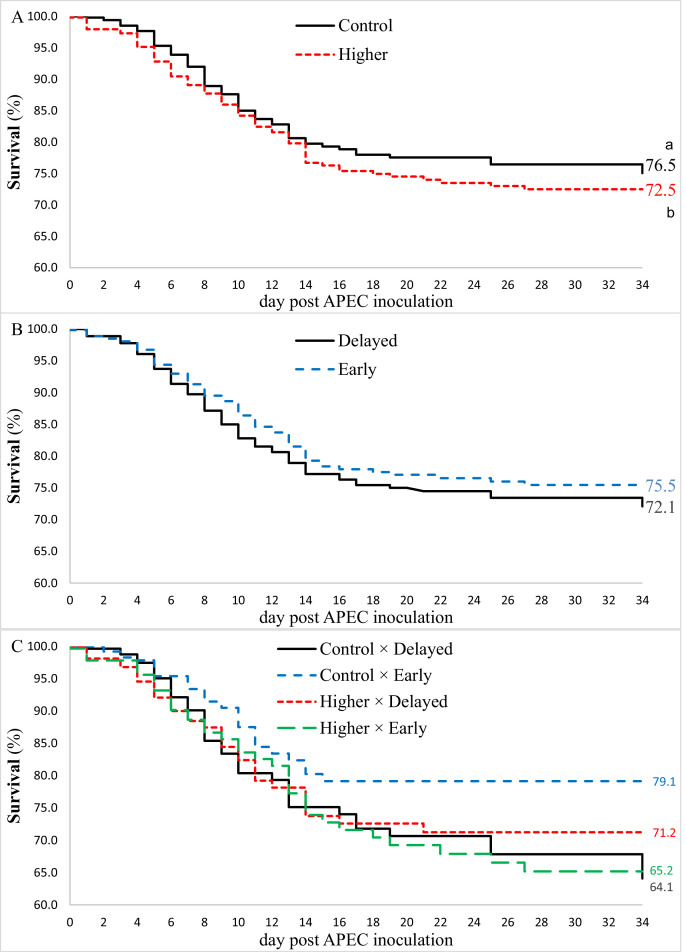
Effect of (A) eggshell temperature (37.8°C [**Control**] or 38.9°C [**Higher**]) during mid incubation (embryo days 7–14) and (B) post-hatch feeding strategy (direct access to feed and water after hatch [**Early**] or 48 h deprivation [**Delayed**]) and (C) interaction between incubation temperature and post hatch feeding strategy on broiler survival probability during 34 d post avian pathogenic *E. coli* (APEC) inoculation at d 8 post hatch. ^a-b^ survival probabilities differ (*P* < 0.05).

First week mortality was low and similar between treatments (total 0.6%). Survival probability p.i. did not show an interaction between EST and feeding strategy (*P* = 0.10; Figure 2C), nor a main effect of feeding strategy (*P* = 0.68; Figure 2B). Survival probability was higher for broilers incubated at control EST compared to higher EST (∆ = 4 %; *P* = 0.04; Figure 2A).

## DISCUSSION

We hypothesized that a higher EST of 38.9°C during mid incubation would accelerate embryo development, improve chick quality at hatch, and consequently enhance the proposed positive effect of early feeding on resilience to colibacillosis more than a control EST of 37.8°C would do. Our findings showed that systemic *E. coli* infection (pericardium and liver) indeed showed an interaction between EST and post-hatch feeding strategy, but opposite to what was expected, whereas local *E. coli* infection (air sacs) was only affected by post-hatch feeding strategy.

A higher incidence of systemic *E. coli* infection was found in early fed broilers compared to delayed fed broilers when incubated at higher EST, whereas at control EST systemic *E. coli* infection was not different between feeding strategies. This finding was expressed by a higher incidence of *E. coli* in blood and a higher incidence of systemic lesions (pericardium and liver) in the higher EST × early feeding compared to the higher EST × delayed feeding treatment groups. Additionally, higher severity of total colibacillosis lesions (tMLS) and worse growth performance in higher EST × early feeding compared to higher EST × delayed feeding was found. This can likely be explained by the higher incidence of systemic *E. coli* infection, because system infection impairs heart and liver function and demands energy to fight the infection that cannot be used for body growth. Wijnen et al. (2022) also found some indications that EST can negatively impact the effect of early feeding. In that study, early fed broilers incubated at a lower EST of 36.7°C during late incubation tended to show higher 1st wk mortality compared to early-fed broilers incubated at control EST. It was speculated that this interaction may have been caused by higher incidence of yolk sac infection due to the combination of worse navel condition in lower EST incubated chicks and an expected higher bacterial load after early feeding. This proposed mode of action cannot explain the interaction between a higher EST during mid incubation and early feeding that was found in the current study. In the current study, no difference in navel condition was found between EST treatment groups and 1st week mortality was low and comparable in all treatment groups (average 0.6%). Only minor differences in chick quality at hatch between EST groups were found in the current study. For instance, YFBM and RY did not differ between EST groups and this is in consistency with previous findings (Wijnen et al., 2020a). Consequently, the effect does not seem to be directly related to chick quality characteristics at hatch and it can only be speculated what biological mechanism caused the interaction that was found in the current study.

Systemic colibacillosis develops when *E. coli* passes through the respiratory tract to the bloodstream and overwhelms the systemic defense (Matthijs et al., 2005). Possibly both a higher EST during mid incubation and early feeding lowered post hatch systemic immune responses. Early feeding may cause long-term immunomodulation via for example alterations in gut microbiota (Brisbin et al., 2008; den Hartog et al., 2016), oral tolerance (Klipper et al., 2001), or fatty-acid metabolism (Cherian, 2015). Studies have shown indications that early fed broilers show lower inflammatory responses than delayed fed broilers (Juul-Madsen et al., 2004; Gonzalez et al., 2011; Ao et al., 2012; Simon et al., 2015). This has not been shown for a higher EST during mid incubation, but higher incubation temperatures from mid incubation onwards resulted in worse developed lymphoid organs at hatch (Oznurlu et al., 2010; Liu et al., 2013; Flores et al., 2016; Leandro et al., 2017). Furthermore, a higher EST applied only during mid incubation altered peripheral blood lymphocyte composition as well as jejunum and bursa morphology at hatch (Wijnen et al., 2020b). These alterations also might have long-term effects, resulting in lower post hatch systemic immune responses. The fact that in the current study a higher EST during mid incubation resulted in a lower survival probability compared to a control EST suggests that a higher EST during mid incubation negatively affected embryo development, although that was not reflected in the chick quality characteristics at hatch, and that post-hatch immunocompetence was impaired. Early feeding could have degraded inflammatory immune responses that were already relatively low after a higher EST during mid incubation, which synergistically resulted in an inadequate response to a systemic *E. coli* infection.

Regardless of EST, early fed broilers showed lower local *E. coli* infections compared to delayed fed broilers, expressed by a lower incidence of local lesions. Probably this caused the tendency for a higher percentage of broilers with no lesions in early compared to delayed fed broilers. Furthermore, early feeding resulted in lower LNvar of standardized body weight deviations during the 13 days p.i. compared to delayed feeding. This indicates that in early compared to delayed fed broilers the negative deviations in body weight due to colibacillosis were either less severe or that recovery was faster or a combination between both (Berghof et al., 2019c; Poppe et al., 2020; van der Zande et al., 2020). The difference in resilience to local *E. coli* infection between feeding strategies may also be the result of a difference in inflammatory response. The inflammatory response seems to play a larger role in the susceptibility to colibacillosis compared to the adaptive immune response as differences in susceptibility are for instance not related to maternal antibodies (Ask et al., 2006b; Ariaans et al., 2008; Dwars et al., 2009; Peng et al., 2018; Alber et al., 2021). As indicated in the previous paragraph, early fed broilers may show lower inflammatory responses compared to delayed fed broilers. Severity of air sac lesions can be explained by inflammatory responses, especially excessive infiltration of macrophages (Dwars et al., 2009; Matthijs et al., 2009), so lower inflammatory responses in early fed broilers than in delayed fed broilers may explain the lower local *E. coli* infection that was found in the current study. In general, the immune system of early fed boilers seems to be ahead in development compared to delayed fed broilers, especially during the first weeks of life (Dibner et al., 1998; Shira et al., 2005; Panda et al., 2010; Hollemans et al., 2020). At first sight, a head start of approximately 48 h in development might seem limited. However, the broiler immune system is immature at the moment of hatch and it develops rapidly during the first week of age (Mast and Goddeeris, 1999; Shira et al., 2003). The first exogenous feed intake after hatch further stimulates this rapid development (Dibner et al., 1998) and consequently, early fed broilers showed higher resilience to local *E. coli* infections compared to delayed fed broilers.

In conclusion, this study was the first to show that EST during mid incubation and post hatch feeding strategy interact on broiler resilience to colibacillosis induced at d 8 of age. A higher EST of 38.9°C during mid incubation in combination with early feeding resulted in worse systemic resilience, whereas at a constant EST of 37.8°C feeding strategies did not differ in resilience to systemic infection. Regardless of EST during mid incubation, early fed broilers had higher resilience to local *E. coli* infections compared to delayed fed conspecifics. Consequently, we recommend the poultry industry to consider early feeding as a strategy to enhance broiler disease resilience to infectious diseases as long as eggs are incubated at the current standard of a constant 37.8°C EST.
